# Masked language modeling pretraining dynamics for downstream peptide: T-cell receptor binding prediction

**DOI:** 10.1093/bioadv/vbaf028

**Published:** 2025-02-20

**Authors:** Brock Landry, Jian Zhang

**Affiliations:** Division of Computer Science & Engineering, Louisiana State University, Baton Rouge, LA 70803, United States; Division of Computer Science & Engineering, Louisiana State University, Baton Rouge, LA 70803, United States

## Abstract

**Motivation:**

Predicting antigen peptide and T-cell receptor (TCR) binding is difficult due to the combinatoric nature of peptides and the scarcity of labeled peptide-binding pairs. The masked language modeling method of pretraining is reliably used to increase the downstream performance of peptide:TCR binding prediction models by leveraging unlabeled data. In the literature, binding prediction models are commonly trained until the validation loss converges. To evaluate this method, cited transformer model architectures pretrained with masked language modeling are investigated to assess the benefits of achieving lower loss metrics during pretraining. The downstream performance metrics for these works are recorded after each subsequent interval of masked language modeling pretraining.

**Results:**

The results demonstrate that the downstream performance benefit achieved from masked language modeling peaks substantially before the pretraining loss converges. Using the pretraining loss metric is largely ineffective for precisely identifying the best downstream performing pretrained model checkpoints (or saved states). However, the pretraining loss metric in these scenarios can be used to mark a threshold in which the downstream performance benefits from pretraining have fully diminished. Further pretraining beyond this threshold does not negatively impact downstream performance but results in unpredictable bilateral deviations from the post-threshold average downstream performance benefit.

**Availability and implementation:**

The datasets used in this article for model training are publicly available from each original model’s authors at https://github.com/SFGLab/bertrand, https://github.com/wukevin/tcr-bert, https://github.com/NKI-AI/STAPLER, and https://github.com/barthelemymp/TULIP-TCR.

## 1 Introduction

This work investigates the masked language modeling method (MLM) of pretraining to predict the binding of antigen peptides to T-cell receptors (TCRs). In the adaptive immune system, antigen peptides are presented to TCRs by a major histocompatibility complex (MHC) molecule. This occurs when the MHC molecule binds to a peptide and presents it on the surface of cells for a TCR to interact with. In short, an immune response occurs when the MHC molecule presents an antigen peptide and the TCR binds to it. Many works employ transformer models to predict this binding interaction for a subset of all possible antigens or TCRs. Specifically, a significant subset of these works attempts to predict the binding of some antigen present in MHC to the complementary determining region 3 (CDR3) α and β chains of a TCR.

Learning to predict the binding of antigen peptides and TCR sequences is important in the modern research fields of cancer immunotherapy and human infectious disease (Grazioli *et al.* 2022). The prediction of peptide:TCR binding can be framed in machine learning as the binary classification of amino acid sequence pairs. Antigen-specific CDR3 chains (α and β) of the TCR are typically <25 amino acids long (Rock *et al.* 1994). Complex chemical properties designate the combinatoric nature and subsequent binding of CDR3 sequences to antigen peptides. The vast problem space created by the unique chemical properties of each amino acid in an antigen peptide or TCR sequence warrants machine learning approaches. To accentuate the complexity of the problem, the TCR repertoire theoretically consists of 10^18^ possible variations (Hou *et al.* 2016). Transformer models have exhibited state-of-the-art performance for peptide-binding prediction problems (Yang *et al.* 2023). The transformer model is a suitable architecture, with much literature surrounding Language Models (LMs). Similar to language data, peptide sequence pairs are often recorded as single-dimensional ordered sequences. More complex representations of amino acid sequence data (SMILES, BLOSUM, etc.) can also be used to include physiochemical properties.

Due to the costs associated with collecting data points of true peptide-binding pairs, unlabeled sequence data are leveraged. In the scenarios pertaining to this work, the *unlabeled* sequence data are used during pretraining to acquire better model weights before learning the downstream task with *labeled* data. MLM is often used to pretrain protein models. MLM is a practice modernized in machine learning by the BERT architecture, used to pretrain transformer models on unlabeled language data (Devlin *et al.* 2019). Unsurprisingly, studies involving pretraining LMs have found that using more pretraining data is generally better (Pérez-Mayos *et al.* 2021). However, the benefit of pretraining for downstream binding prediction performance also depends on the relevance of the pretraining corpus to the downstream task (Gururangan *et al.* 2020).

The following sections will explain the complete training process of three cited works (Wu *et al.* 2021, Kwee *et al.* 2023, Myronov *et al.* 2023) and show the downstream performance of their prediction models after pretraining intervals. Identifying an optimal amount of pretraining (or lack thereof) motivates the analysis. During replication of these models and our own similarly designed models, we observed that converged pretraining does not translate to higher downstream performance. Therefore, selecting the best-performing (lowest training/evaluation loss) pretrained model checkpoint does not simply yield superior downstream performance. For each model observed, the downstream performance gain begins to oscillate around a mean, substantially before the pretraining converges. This is in contrast to the intuition that better pretraining performance would yield better downstream performance. This work demonstrates a point of significantly lower returns before pretraining converges, where higher pretraining accuracy and lower loss metrics do not directly translate to downstream performance benefits. The results demonstrate a point of *sufficient* pretraining, where no guaranteed downstream performance benefit is obtained from increasing the MLM pretraining performance. In short, complete mastery of this pretraining task does not translate to higher performance in the finetuning task.

It is important to note that the models produced in this work are derived from BERT architectures of similar sizes pretrained on data that were aggregated and curated with similar methods. These are small language models (SLMs) with 40.8 M or 110 M total parameters. The pretraining corpora used in these models are generally smaller than those used in larger protein models, such as TAPE (Rao *et al.* 2019) or ESM-1b (Rives *et al.* 2021). These larger protein models pretrain on broader categories of longer protein sequences, instead of short antigen and CDR sequences (which are used in this work). Although impractical to compare empirically, these architectural and data distinctions are acknowledged. The findings of this work are also not indicative of protein models with different learning curriculums. This work specifically pertains to BERT-like SLMs that perform MLM pretraining on short peptide sequence data and finetune for similar peptide downstream binding prediction tasks. The specifications of the models and data corpora will be further elaborated on in Sections 2 and 3. The observations in this work are not expected to be replicated in dissimilar problem settings.

In the work below, Section 2 explains the necessary background information for the MLM pretraining process and transformer architectures. Section 3 explains the distinctions between the pretraining and finetuning procedures for each pretraining process studied in this work. In Section 4, we exhibit the downstream performance achieved from many intermittently saved model states (or pretrained model checkpoints) acquired successively during pretraining. The downstream performance for each pretrained model checkpoint is examined against its own MLM performance metrics.

## 2 Methods

### 2.1 Data collection

Modern sequencing technologies have led to an increase in the availability of sequence data in recent decades. The resulting databases have grown exponentially, with the total number of sequences doubling every 2 years (Cheng *et al.* 2021). Each pretraining scenario for the models studied in this work uses different datasets for their pretraining. Each dataset compromises sequence relevance and the overall breadth of sequences for the finetuning or downstream objective.

While positive-labeled peptide-binding samples are scarce, negative-labeled peptide-binding samples are often generated with a reasonably high accuracy (Myronov *et al.* 2023). These negative binding pairs are generated by mismatching recorded positive peptide-binding samples. The peptide:TCR datasets used in this work were all obtained from existing public sources (Wu *et al.* 2021, Kwee *et al.* 2023, Meynard-Piganeau *et al.* 2023, Myronov *et al.* 2023). The specifics of these sources will be discussed in Section 3.

### 2.2 Masked language modeling

It is commonly observed that pretraining machine learning models can be valuable in cases where finetuning data is limited (Cheng *et al.* 2021). As a standard of BERT architectures, the input sequences (antigen peptides and TCRs) are represented as a sequence of tokens. In the application of peptide:TCR binding prediction, the token vocabulary consists of the 20 human-occurring amino acids and additional special tokens to designate the starting position of the sequence, the end of the sequence, the mask, and the padding. When classifying a peptide:TCR pair, the two sequences are concatenated into a single sequence of tokens with the additional special tokens that were described previously. Positional information is generated to contextualize intrasequence amino acid placement and also designate each amino acid as either within the antigen or TCR portion of the sample. The MLM pretraining method learns an embedding for each token. During forward propagation, the token embedding exits the embedding and transformer layers to be passed to a token classification head during pretraining or a binding classification head during finetuning.

The models in this study employ a nearly identical BERT-like MLM pretraining scenario. Specifically, 15% of tokens are selected for prediction, and the training objective is to predict the selected token given its context in the broader sequence. Before prediction, the selected token is replaced with a MASK token in 80% of instances, replaced with a random token in 10% of instances, or not replaced in the remaining 10% of instances. This MLM pretraining procedure has been emulated in many transformer models that predict peptide sequence interactions (Rao *et al.* 2019, Cheng *et al.* 2021, Wu *et al.* 2021, Kwee *et al.* 2023, Meynard-Piganeau *et al.* 2023, Myronov *et al.* 2023). Large amounts of unlabeled peptide sequence data can be used during MLM pretraining to increase downstream peptide-binding prediction. The guiding intuition is that a valuable intermediate contextual embedding can be achieved from MLM and applied to subsequent downstream tasks.

MLM pretraining is ultimately convenient due to the greater availability of unlabeled peptide sequence data compared to labeled data. Considering the allure of MLM pretraining and the accessibility of unlabeled data, the complete training curriculum often results in increased training time and energy expenditure. In the scope of this work, each model’s final performance is the result of significantly longer computational time spent pretraining than finetuning.

### 2.3 Peptide:T-cell receptor binding prediction

After pretraining, a separate sequence classification head of the transformer model is trained to perform binary classification of the labeled input sequences. In the scope of this work, peptide:TCR binding prediction is the downstream task performed after MLM pretraining. Predicting the binding classification on labeled peptide sequence binding data is the finetuning task for each model. Some works choose to “freeze” the layer weights derived from pretraining and only perform gradient descent on the weights in the classification head. This freezing can be useful, especially in the scenario where multiple classification heads are to be applied to the same pretrained model. In the scope of this work, the layer weights are not frozen and are therefore updated during the finetuning process. The hidden representation of the sequence, learned during both pretraining and finetuning, is passed into the classification head. Typically, the classification head is a feedforward neural network layer with output neurons equal to the number of class labels. In the case of binary classification, it is typically a single output neuron preceding a sigmoid activation function. For each model training curriculum replicated in this work, finetuning consumes a minority of the overall training cost due to the proportion of labeled binding pairs to unlabeled pairs used during pretraining.

## 3 Implementations

This work investigates three specific transformer architectures to identify the efficacy of their loss-driven pretrained model selection. These models are BERTrand (Myronov *et al.* 2023), TCR-BERT (Wu *et al.* 2021), and STAPLER (Kwee *et al.* 2023). Each of the models uses MLM pretraining to increase downstream performance for peptide-binding prediction. These models leverage MLM pretraining in the absence of surplus labeled data, in order to increase downstream binding prediction performance. The benefit of MLM pretraining in this scenario has yielded a downstream performance benefit in many prior works (Cheng *et al.* 2021). For simplicity, each of the models investigated in this work use PyTorch and the Huggingface transformers library to implement BERT-like architectures.

The models investigated in this work are BERT networks with 8 and 12 transformer blocks. Other notable dimensions are expressed in Table 1. The BERTrand and STAPLER models are designed to classify the binding of any peptide:TCR input sequence pair that meets the size constraints of the input layer. Notably, the model performance is largely impacted by the focus of the finetuning dataset; therefore, it is unlikely to have comparable performance across all peptide:TCR binding scenarios. The TCR-BERT model uses only a TCR sequence as input because it is trained to classify any TCR sequence against only one single antigen.

**Table 1.** Transformer model dimensions including the amount of layers, hidden units, and self-attention heads.

Model	Layers	Hidden units	SA heads
BERTrand	8	512	8
TCR-BERT	12	768	12
STAPLER	8	512	8

In our tests, increasing the size of the model did not increase the performance of MLM or the downstream performance. However, a substantial decrease in the model dimensions would have a negative impact on MLM and downstream performance. This negative impact was clearly observed for each model when their dimensions were decreased to 4 layers, 256 hidden units, and 4 self-attention heads. These results were not systematically analyzed because of the additional computational costs and time associated. Furthermore, this study intends to use the unmodified version of each published model included for ease of reproducibility. The dimensions of each model are included in Table 1.

As described above, the pretraining datasets for each model contain CDR3 sequences. Antigen peptide sequences are also present in the BERTrand and STAPLER pretraining corpus. The specific number of sequences in each pretraining corpus is shown in Table 2.

**Table 2.** This table displays pretraining data quantities for each model.^a^

Model	Antigens	CDR3s	Total PT Samples
BERTrand	150k	560k	11m
TCR-BERT	0	89k	89k
STAPLER	183k	160k	32m

a Notably, the STAPLER pretraining corpus is randomly generated after each epoch by pairing each CDR3 sequence to a random antigen sequence. BERTrand and TCR-BERT both have a static pretraining corpus, which is determined before pretraining.

In order to analyze the similarity of the pretraining and finetuning datasets for each model, the Needleman–Wunsch algorithm (via the Python pairwise sequence alignment module) was used. The CDR3b sequences in each pretraining dataset were analyzed against the CDR3b sequences in the corresponding finetuning dataset. The same was done for the antigen sequences of each model. The results are displayed in Table 3.

**Table 3.** Sequence alignment percentages and scores are exhibited for each model.^a^

Metric	BERTrand	TCR-BERT	STAPLER
Antigen score	4.5		7.1
Antigen percent	15.2%		22.6%
CDR3b score	21.5	22.6	22.1
CDR3b percent	47%	50.5%	49.4%

a The needle (Needleman–Wunsch algorithm) alignment algorithm was applied to random sequence pairs from the pretraining and finetuning datasets for each model.

### 3.1 BERTrand

In the original BERTrand work, the model is pretrained on a corpus of 9 m training sequence pairs and 2 m evaluation sequence pairs. The BERTrand scripts assemble the corpus with 11 m (unpaired) CDR3β sequences and 150 k antigen peptides presented by MHC-I molecules. 11M CDR3β sequences were obtained by simulated VDJ recombination of 85 healthy donors using immuneSim (Dean *et al.* 2015, Cheng *et al.* 2021), and 150 k peptides were acquired from the results of mass spectrometry peptide sequencing experiments (Abelin *et al.* 2017). To generate the corpus, the antigen peptides are randomly matched with the CDR3β sequences. Thus, each unlabeled pretraining sample is composed of a random short antigen peptide and a CDR3β sequence.

**Figure 1.**
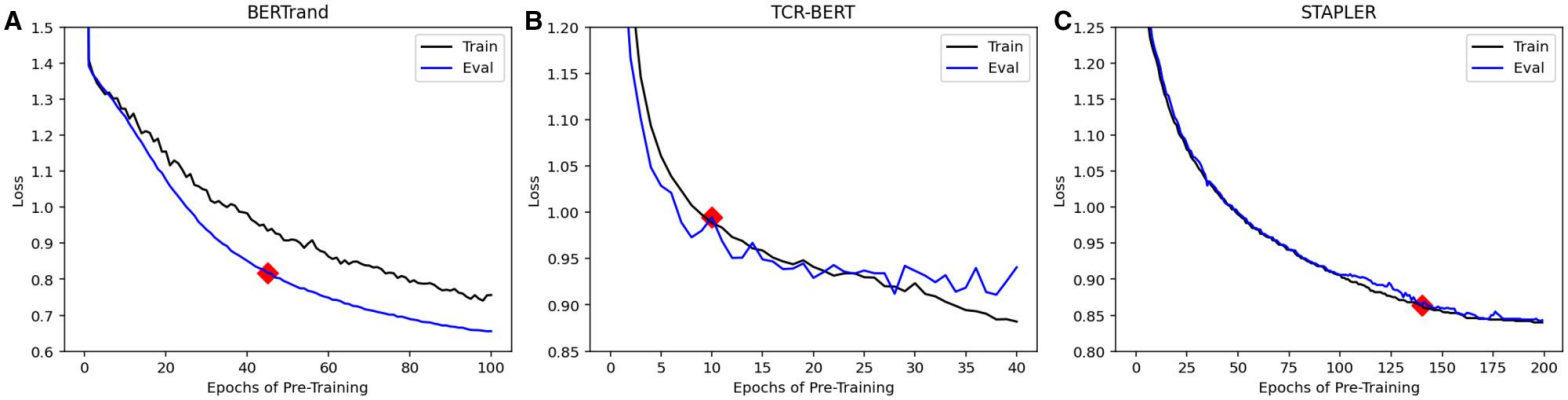
This figure exhibits the pretraining loss curves for each model studied. The best downstream-performing pretrained model checkpoint is highlighted in red on each loss curve. (A) The training and evaluation loss of the BERTrand model is displayed for 100 epochs of MLM pretraining. (B) The training and evaluation loss of the TCR-BERT model is displayed for 30 epochs of MLM pretraining. (C) The training loss of the STAPLER model is displayed for the 200 epochs of MLM pretraining.

When using the unedited pretraining scripts for the BERTrand model, the pretraining duration is for 100 epochs that require over 19 days on a single NVIDIA RTX 3090 GPU. The pretraining loss and evaluation loss are exhibited in Fig. 1. The program of Myronov *et al.* (2023) uses the pretrained model checkpoint that performed best on the pretraining evaluation data. When replicating the study, the highest performing model checkpoint was obtained during pretraining after 99 epochs. After pretraining, there are three datasets used for finetuning. These datasets include non-cancer and known cancer peptide-binding pairs. The model is finetuned on 21 cross-validation splits for each dataset, which amount to 63 total rounds of cross-validation.

### 3.2 T-cell receptor-BERT

In the original TCR-BERT (Wu *et al.* 2021) training process, the model is pretrained using BERT-like MLM on a pretraining corpus of 88 576 CDR3α and CDR3β sequences from the VDJdb (Bagaev *et al.* 2020) and PIRD (Zhang *et al.* 2020) datasets. This relatively small pretraining corpus trains much faster than the BERTrand corpus, requiring only 3 h of pretraining time for 30 epochs of MLM on a single NVIDIA RTX 3090 GPU.

In particular, this model differs from the others in this work because the CDR3β sequences are the lone input to the model. Each CDR3β sequence is classified against one antigen rather than any antigen with which it is paired. Therefore, binary classification is based on whether a given CDR3β sequence binds to one specific antigen for which the model is trained. Although limited, this methodology can offer high prediction accuracy for specific antigens. This higher prediction accuracy is due to the fact that the problem structure is greatly simplified. Models trained in this way can be useful for designing TCR sequences that bind to the antigen of concern. Alternatively, BERTrand and STAPLER perform binary classification on any sequence pair (antigen *and* CDR3) that is in accordance with the model’s input constraints.

Unfortunately, the finetuning dataset used to achieve the results in the TCR-BERT paper (Wu *et al.* 2021) was not included in their GitHub repository. Lacking the exact methodologies to recreate the finetuning dataset in the code-base from Wu *et al.*, a dataset was generated for the purpose of analyzing the pretraining performance. This finetuning dataset of 26 k labeled CDR3β sequences was generated with sequences observed in healthy human donors. The source data were obtained from (Meynard-Piganeau *et al.* 2023). After pretraining, the model was finetuned with five different random splits of cross-validation data.

### 3.3 STAPLER

The STAPLER model is pretrained using standard MLM on a pretraining corpus of 159 859 CDR3 sequences (α and β) and 183 398 peptide sequences known to bind to MHC-I molecules. The dataset was assembled by Kwee *et al.* (2023) from multiple studies (Yost *et al.* 2019, Wu *et al.* 2020, Zheng *et al.* 2021, Kourtis *et al.* 2022, Liu *et al.* 2022). Each pretraining epoch randomly pairs the 159 859 CDR3 sequences with one antigen sequence each. Random pairing is used to generate unique batches in each epoch, increasing the complexity of the pretraining task. These pairs can largely be assumed to be nonbinding pairs. Lacking true binding pairs during MLM pretraining is not consequential, because MLM does not learn binding classification. Alternatively, the finetuning corpus does not randomly mismatch the binding pairs since the correctness of the pairings is paramount to the learning task.

In the original scripts, the STAPLER model is pretrained for 500 epochs. However, when replicating the work, the original training parameters listed in the paper and GitHub did not train comparably to what was reported in the work. In particular, the learning rate, weight decay, and number of epochs were minorly adjusted to produce better pretraining results. These modifications resulted in pretraining that yielded a similar performance to what was originally reported by Kwee *et al.* (2023). After our adjustments, comparable loss convergence occurred around 200 epochs, with the highest recorded pretraining performance occurring at the 195th epoch. All 200 pretraining epochs required 12 h to complete on a single NVIDIA RTX 3090 GPU. After pretraining, the model was finetuned on five different cross-validation splits of the finetuning dataset.

## 4 Results

This section exhibits the MLM pretraining loss curves and downstream performance curves of the three previously described peptide:TCR binding prediction models. The downstream performance is evaluated for discrete intervals of pretraining. Each model is pretrained using the original scripts until the pretraining loss converges. Afterward, the finetuning exercise was repeated five times for each model checkpoint, and the average downstream performances were recorded.

### 4.1 Pretraining loss evaluation

Each binding prediction model described in the previous section was replicated and pretrained until the training loss converged. Figure 1 displays the loss curve recorded during each model’s pretraining process. The pretraining process for each model follows a typical training and evaluation loss trajectory, with the lowest loss metrics occurring near the final epoch.

**Figure 2.**
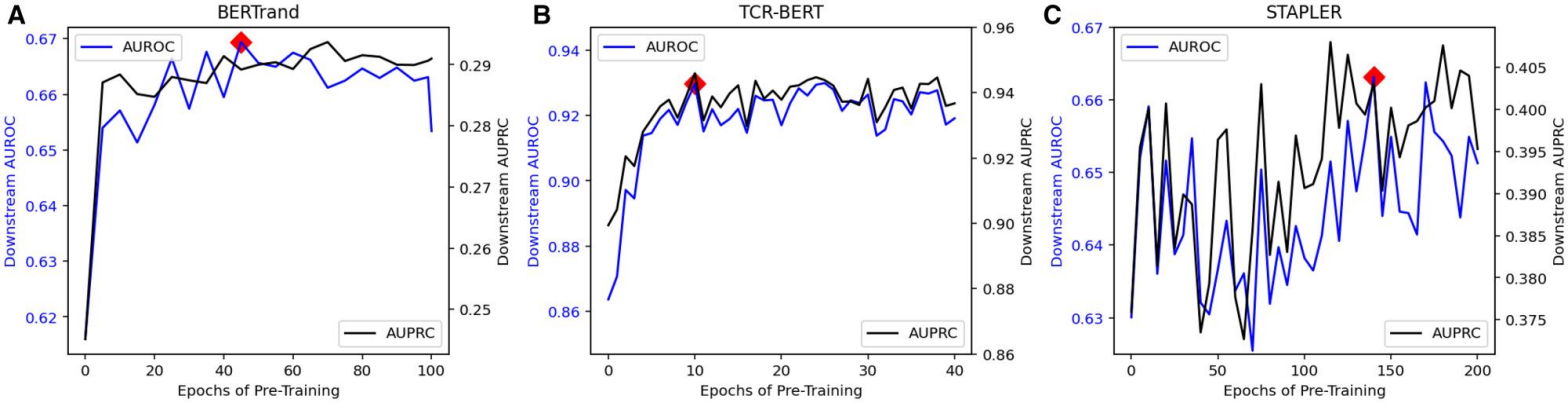
(A) Downstream performance of the BERTrand model is displayed for 100 epochs of MLM pretraining. Highlighted on the curve, Epoch 45 resulted in the highest average downstream performance between the cancer and non-cancer datasets after 21-splits of finetuning. (B) Downstream performance of the TCR-BERT model throughout 30 epochs of MLM pretraining is displayed. Highlighted on the AUPRC curve, Epoch 10 resulted in the highest average downstream performance between the cancer and non-cancer datasets after 10-splits of finetuning. (C) Downstream performance of the STAPLER model throughout 200 epochs of MLM pretraining is displayed. Highlighted on the curve, the pretraining checkpoint after 140 epochs resulted in the highest average downstream performance across five splits of finetuning.

BERTrand’s highest performing epoch during pretraining was Epoch 99, but Epoch 45 is highlighted on the loss curve because it resulted in the highest downstream performance. The downstream performance metrics will be elaborated on in the next section and in Fig. 2. TCR-BERT follows a similar pattern, where Epoch 28 has the best pretraining loss metrics, but Epoch 10 has higher downstream performance albeit its inferior loss metrics. Finally, STAPLER’s training loss is the lowest at Epoch 195 with Epoch 140 resulting in the highest downstream performance.

### 4.2 Downstream performance evaluation

The original scripts used for the BERTrand implementation performed a downstream peptide-binding prediction task with 21 cross-validation splits on each of three finetuning datasets. The 63 total splits require 25 h on the RTX 3090 GPU for each model checkpoint. Due to time constraints, we chose to finetune the first of every five pretrained checkpoints for all 63 splits of the finetuning data. The evaluation is shown in Fig. 2. This evaluation concluded that the 45-epoch checkpoint was the highest performing pretrained model, with the highest average AUROC across 63 training splits on the unseen testing data (cancer and non-cancer).

The finetuning dataset used for the TCR-BERT model was much smaller; therefore, *each* pretrained model checkpoint was finetuned for five randomly paired splits of the finetuning data. Although only pre-trained for 30 epochs, TCR-BERT’s downstream performance curve quickly peaks with the 10th epoch checkpoint transferring the highest downstream performance. To be clear, the performance metrics of this model are higher than those shown in the original TCR-BERT publication. The increased performance is due to the alternate finetuning dataset used in this study. The original dataset is not supplied in the authors GitHub or paper, see Section 3.2.

Similar to BERTrand, we only tested one out of every five checkpoints from the STAPLER model’s 200 pretrained model checkpoints. The finetuning process for five splits of training data required 9 h of compute time on the RTX 3090 GPU. The STAPLER implementation conducts the MLM training exercise on the finetuning dataset. This additional MLM training is often called Task Adaptive PreTraining (TAPT) (Gururangan *et al.* 2020). The TAPT method is probably a causal factor in the shape of the downstream performance curve shown in Fig. 2. The downstream performance gained from pretraining approaches peak values much faster than the BERTrand and TCR-BERT models. However, the STAPLER model’s highest performing pretraining epoch is produced relatively later in the pretraining process than the previous models. The oscillations can likely be attributed to gradient overshooting during finetuning. Alternatively, there may be additional payoff gained from the random creation of samples during pretraining, resulting in a more dynamic pretraining scenario than BERTrand and TCR-BERT. With that being stated, similar to the BERTrand and TCR-BERT models, the STAPLER model yields multiple pretrained model checkpoints with higher downstream performance than the checkpoint with the best loss metric (Epoch 195).

Summarized in Table 4, each work yields a pretrained model checkpoint with the highest downstream performance, occurring significantly before the pretraining loss converges. These findings indicate that achieving pretraining loss convergence can be an unnecessary or imperfect objective in similar peptide-binding prediction models. The downstream performance curve converges substantially before the pretraining loss curve.

**Table 4.** Pretraining loss and downstream AUROC of the highest performing pretrained models.

Model	Best epoch	AUROC	Loss
BERTrand	45/100	0.669	0.818
TCR-BERT	10/30	0.9297	0.9945
STAPLER	140/200	0.4942	0.864

After the pretraining loss nears 1.0, variations in the gradient overshoot yield the highest performing pretrained model checkpoints. This observation is most stark when viewing the BERTrand performance metrics. The BERTrand model’s downstream performance nearly peaks with only 25 pretraining epochs, in which the evaluation loss equals 1.0. The pretraining evaluation loss reaches as low as 0.65 in Epoch 99, but does not yield any additional downstream improvement.

Each model’s downstream performance curve has slightly different skews, but all reach a convergence point relatively early in the pretraining. Beyond this point, further pretraining does not offer any certain benefit. The loss threshold of 1.0 is a heuristic value used to demonstrate the diminishing returns of further pretraining. This value of 1.0 was chosen because of its speed of acquisition, low training commitment required and its observed impact on the downstream performance curves. For each scenario, after the training loss became lower than 1.0, the downstream performance of subsequent pretrained model checkpoints appears to be subject to variance incurred by gradient overshoot oscillations, rather than any traceable increase (shown in Fig. 2).

Table 5 shows the mean AUROCs and standard deviations for each model checkpoint at which the pretraining loss is below 1.0. For both the BERTrand and TCR-BERT models, the highest performing model does not exceed the average performance by more than 2 SD. The highest performing STAPLER model exceeds the average by nearly 3 SD. With that being stated, these standard deviations are minuscule, and the overall performance curve is relatively flat for each model beyond the loss threshold of 1.0.

**Table 5.** Performance analysis of the pretrained models after surpassing a loss of 1.0.^a^

Model	Epochs	Mean AUROC	AUROC STD
BERTrand	25 – 100	0.664	0.003
TCR-BERT	7 – 30	0.9228	0.0046
STAPLER	45 – 200	0.4723	0.0075

a The Epochs column indicates the range of epochs, which achieved a loss better than 1.0. The mean and standard deviation of the downstream performance resulting from finetuning each of these epochs’ saved state are tabulated accordingly.

## 5 Discussion

Many model architectures in the literature have shown a clear downstream performance benefit from employing MLM (Rao *et al.* 2019, Cheng *et al.* 2021, Wu *et al.* 2021, Kwee *et al.* 2023, Meynard-Piganeau *et al.* 2023, Myronov *et al.* 2023). MLM pretraining with transformer models has proven to be a useful precursor for peptide:TCR binding prediction tasks when the amount of relevant pretraining data greatly exceeds the finetuning data. It is clear that each model observed in the results section benefits from just a single epoch of pretraining.

This work was motivated by a notable amount of variation in the pretraining scenarios seen in published peptide:TCR binding prediction models. Similar to the binding prediction models studied in this work, there are many other works which pretrain until loss convergence. The trend observed in our results section indicates that around a loss metric of 1.0, the downstream performance appears mainly subject to minor gradient overshoots with no clear benefit observed from additional pretraining. The model weights obtained from each pretraining epoch are likely to have minor variability in the trickle-down effect they impart on the downstream performance.

Based on our analysis, the pretrained model checkpoint, which transfers best to the finetuning task, is likely to score lower than a 1.0 evaluation loss during pretraining. Different pretraining and finetuning datasets are expected to alter the trend of the performance metrics observed in this work. Specifically, a much larger and more diverse pretraining corpus will likely exhibit a downstream performance trend that differs from those in this study. Unfortunately, any substantial increases in the size of the pretraining corpus would incur great energy and computational costs to study in a similar fashion.

Although MLM pretraining provides a fundamental learning task for the model, there is a consequential distinction between the pretraining and finetuning task. The pretrained embeddings generated after each epoch are not optimized to directly address the finetuning task, they are optimized to predict missing amino acids in the context of a broader amino acid sequence. This information is of course transferrable to the downstream task, but the results of this work demonstrate that increasing the model’s MLM performance does not indefinitely provide a linear increase to the downstream peptide-binding prediction performance. The training curriculum studied in this work appears to be loosely analogous to learning the alphabet (pretraining) before learning to read (finetuning). Only a working understanding of the alphabet is necessary to learn to read, whereas further mastery of the alphabet (e.g. memorizing each letter’s index or the reverse-ordering) is not helpful for the task of reading.

To summarize, the results demonstrate that when the pretraining loss reaches 1.0, the downstream peptide:TCR binding performance benefits are nearly diminished or are completely diminished. Beyond this point for each subsequent pretrained model checkpoint observed, the downstream performance resulted in bilateral deviations from a mean downstream performance. These tests do not encapsulate the infinite spectrum of peptide pretraining and finetuning scenarios, but the results strongly suggest that the most pretrained model checkpoint is not likely to proportionally benefit downstream performance. Therefore, achieving higher MLM pretraining performance to the point of loss convergence may be a counterproductive focus when the end goal is solely to increase downstream performance. In the scenarios observed in this work, the best performing models during pretraining did not produce the best downstream performance. The downstream performance benefit accrued from MLM pretraining does not follow the conventional behaviors associated with overfitting and underfitting observed in the finetuning task.

Observing the downstream performance of each pretrained model checkpoint is an expensive method that may not be feasible for every research scenario. Although this work does not provide a definitive way to cheaply select the best pretrained model checkpoint, it demonstrates an often wasteful practice that exists in the relevant literature. The practice of selecting a pretrained model checkpoint based solely on loss metrics yields a variable impact on downstream performance when replicated. Our intention in this work is to provide a clear understanding of the observed pretraining dynamics, so that it can be applied to future peptide:TCR binding prediction studies.
